# Incidence, predictors, and outcomes of new‐onset atrial fibrillation in patients with ST‐elevation myocardial infarction

**DOI:** 10.1111/anec.12746

**Published:** 2020-01-23

**Authors:** Mohamed Khalfallah, Ayman Elsheikh

**Affiliations:** ^1^ Department of cardiovascular medicine Faculty of Medicine Tanta University Tanta Egypt

**Keywords:** incidence, new‐onset atrial fibrillation, outcomes, predictors, STEMI

## Abstract

**Background:**

Atrial fibrillation (AF) is a common arrhythmia and one of the complications in the setting of ST‐elevation myocardial infarction (STEMI). Our objective of the present study was to investigate the incidence, predictors, and outcomes of NOAF in patients with acute STEMI managed with pharmacoinvasive strategy (PIS) versus those managed with primary percutaneous coronary intervention (PPCI).

**Methods:**

The study included 530 patients with STEMI divided into two groups according to the method of treatment. Group I: 269 patients subjected to pharmacoinvasive strategy (PIS), group II: 261 patients managed with primary percutaneous coronary intervention (PPCI). Incidence, predictors, and outcomes of NOAF were assessed in each group separately.

**Results:**

The incidence of NOAF was 25 patients (9.3%) in group I and 24 patients (9.2%) in group II. Multivariate regression analysis identified the independent predictors of NOAF that were (advanced age ˃65 years, history of hypertension, left atrial volume index (LAVI) ˃34 ml/m^2^, E/e’ ratio ˃ 12, right coronary artery (RCA) as a culprit vessel and presence of heart failure). There was no statistically significant difference between both groups regarding the occurrence of MACE.

**Conclusion:**

New‐onset AF represents one of the common complications in the setting of STEMI. Advanced age, hypertension, LAVI ˃34 ml/m^2^, E/e’ ratio ˃12, RCA culprit vessel, and heart failure were the independent predictors of NOAF.

## 1 INTRODUCTION

Atrial fibrillation (AF) is one of the commonest arrhythmias occurring in the setting of acute myocardial infarction (AMI) with incidence varying from 5% to 18% (Pizzetti et al., 2001; Sugiura et al., 1985). Notably, the incidence of AF has declined to reach 4.8%–7.7% in the setting of AMI in the era of primary percutaneous coronary intervention (PPCI) (Gurm et al., 2008; Kinjo et al., 2003; Lopes et al., 2009, 2008). The occurrence of new‐onset AF (NOAF) after ST‐segment elevation myocardial infarction (STEMI) is associated with increased morbidity and mortality of such patients (Lehto, Snapinn, Dickstein, Swedberg, & Nieminen, 2005; Rathore et al., 2000). In the developing countries, the ideal management of patients with STEMI is pharmacoinvasive strategy (PIS) as PPCI is not always available, especially if the patient admitted to a non‐PCI capable center. PIS is defined as pharmacological reperfusion followed by rapid transfer for coronary angiography and PCI within 3–24 hr (Capodanno & Dangas, 2012; Khalfallah, Elsheikh, & Abdalaal, 2018; Mrdovic et al., 2012). The treatment strategy may affect the incidence or outcomes of certain diseases Khalfallah, Abdalaal, and Adel (2019), so our objective of the present study was to investigate the incidence, predictors, and outcomes of NOAF in patients with acute STEMI, as the previous studies Kinjo et al. (2003) and Mrdovic et al. (2012) investigated NOAF in patients with STEMI who were managed with PPCI. In the present study, we selected a group of patients managed with PIS and another group managed with PPCI. Moreover, for the best of our knowledge this is the first study that comparing the two strategies of treatment to investigate if there is a difference in incidence, predictors, or outcomes of NOAF in patients with STEMI between the two strategies of treatment.

## 2 METHODS

This prospective study included 530 patients who were presented to our cardiology department, with STEMI during the period from June 2017 to January 2019. They were classified into two groups according to the method of treatment: Group I: 269 patients received fibrinolytic therapy in surrounding non‐PCI capable centers and referred to our department for PIS, with further subdivision into two subgroups; group IA (Patients with sinus rhythm) and group IB (Patients with NOAF). Group II: 261 patients with STEMI admitted directly to our cardiology department and managed with PPCI, with further subdivision into two subgroups; group IIA (Patients with sinus rhythm) and group IIB (Patients with NOAF). The study was approved by local ethical committee, and all patients participated in the study signed a written informed consent. Every patient had a code number pointed to his name, address, and telephone number, and these data were saved in a special file.

Exclusion criteria: patients with STEMI presented later after the first 24 hr, patients with known history of permanent AF, and patients who still in AF after trial of cardioversion.

All patients were subjected to full detailed history taking and clinical examination with stress on coronary artery disease risk factors; diabetes mellitus, systemic hypertension, dyslipidemia, and smoking. History was taken about the occurrence of previous AF or other arrhythmias and those patients were excluded from the study. Electrocardiogram (ECG) was done, and the rhythm was checked for the presence of AF. NOAF was defined as ECG evidence of irregular sustained rhythm with no evidence of discrete atrial activity in patients with negative history of persistent or permanent AF (Windecker et al., 2014). Also, patients were monitored during hospital stay and any observations of rhythm changes were registered and patients with NOAF were included in the study. The duration of AF was measured and registered. Blood samples were extracted for routine laboratory tests. All patients were premedicated with dual antiplatelet therapy and received the standard medical treatment including beta blockers, angiotensin‐converting enzymes inhibitors, statins, and anticoagulation therapy according to the guidelines and the status of every patient, and admitted to cardiac catheterization. Diagnostic coronary angiography was done, PCI procedural access was femoral or radial approach according to the operator judgment, the radial approach was the preferred access to minimize the risk of access site bleeding especially in PIS after fibrinolytic therapy, the culprit lesion was determined and assessed for the site, thrombus burden and TIMI flow, balloon predilatation was done and a stent or stents were deployed according to operator judgment. Patients received the proper medications after the procedure according to the recent guidelines (Windecker et al., 2014). Echocardiography was performed after the procedure using Vivid 7 (GE Healthcare, Horten, Norway) with determination of left ventricular ejection fraction (LVEF) using the Simpson's biplane method. Left atrial volume index (LAVI) was calculated from apical four‐ and two‐chamber views. E/e’ ratio was determined in all patients.

The occurrences of major adverse cardiac events (MACE) in the form of (cardiac death, reinfarction, cardiogenic shock, heart failure, major bleeding, and cerebral stroke) were reported. The patients were followed during hospital stay for recovery of AF and cardioversion either by medical treatment in the form of intravenous amiodarone or DC shock was tried according to the medical status of the patient and recent guidelines. After discharge, the patients were followed up for three months for the occurrences of MACE and the recurrence of AF.

### 2.1 Statistical analysis

Statistical analysis was done using SPSS version 23. Baseline data were expressed as mean ± standard deviation (*SD*). Categorical variables were expressed as numbers and percentages. Student's *t* test was used to check the differences between the two groups in quantitative data. Chi‐square (*χ* ^2^) test was used in order to compare proportions between two qualitative parameters. *p* value <.05 was considered statistically significant. Multivariate regression analysis was used to identify the independent predictors of NOAF.

## 3 RESULTS

The study included 530 patients with STEMI. The patients were divided into two groups. Group I (PIS group) included 269 patients while group II (PPCI group) included 261 patients. The basal characteristics, echocardiographic data, and angiographic results of the 2 groups were shown in Table 1. The incidence of NOAF was 25 patients (9.3%) in group I versus. 24 patients (9.2%) in group II with *p* value = .969. There was no statistically significant difference between the two groups regarding the age of the patients, risk factors for NOAF and echocardiographic parameters. The angiographic results, mortality and the occurrence of MACE showed no statistically significant difference between the two groups. The duration of AF was measured, and its mean was (6.51 ± 5.53) hours.

**Table 1:** Basal characteristics, echocardiographic data, angiographic results, and major adverse cardiac events in both groups

	Group I (*n* = 269) (PIS group)	Group II (*n* = 261) (PPCI group)	*p* value
Age, years	62.35 ± 9.40	62.25 ± 9.76	.904
Male gender, *n* (%)	148 (55%)	150 (57.5%)	.569
Smoking, *n* (%)	46 (17.1%)	61 (23.4%)	.072
Hypertension, *n* (%)	94 (34.9%)	72 (27.6%)	.068
Diabetes mellitus, *n* (%)	108 (40.1%)	112 (42.9%)	.473
Dyslipidemia, *n* (%)	119 (44.2%)	102 (39.1%)	.229
Prior MI, *n* (%)	23 (8.6%)	33 (12.6%)	.125
NOAF, *n* (%)	25 (9.3%)	24 (9.2%)	.969
LVEF, (%)	46.32 ± 4.06	45.64 ± 6.18	.138
LAVI, ml/m^2^	33.70 ± 1.47	33.49 ± 2.02	.174
E/e’ ratio	11.39 ± 1.26	11.58 ± 1.39	.103
LM coronary artery, *n* (%)	4 (1.5%)	4 (1.5%)	.966
LAD coronary artery, *n* (%)	106(39.4%)	119(45.6%)	.150
CX coronary artery, *n* (%)	74(27.5%)	86(33%)	.173
Right coronary artery, *n* (%)	73(27.1%)	63(24.1%)	.429
Mortality, *n* (%)	7 (2.6%)	5 (1.9%)	.595
Cardiogenic shock, *n* (%)	18 (6.7%)	29 (11.1%)	.074
Heart failure, *n* (%)	22 (8.2%)	21 (8.0%)	.955
Major bleeding, *n* (%)	8 (3.0%)	4 (1.5%)	.265
Reinfarction, *n* (%)	5 (1.9%)	8 (3.1%)	.369
Cerebral stroke, *n* (%)	3 (1.1%)	4 (1.5%)	.674

Abbreviations: CX, circumflex; LAD, left anterior descending; LAVI, left atrial volume index; LM, left main; LVEF, left ventricular ejection fraction; MI, myocardial infarction; NOAF, new‐onset atrial fibrillation.

Subgroup analysis of group I (PIS group) showed that patients with NOAF (subgroup IB) were advanced in age than patients who remained in sinus rhythm (subgroup IA). Smoking and hypertension as risk factors were more frequent in subgroup IB. Echocardiographic characteristics showed that patients in subgroup IB had a lower LVEF, higher E/e’ ratio, and higher LAVI than patients who remained in sinus rhythm. The angiographic results showed that, the right coronary artery (RCA) as the culprit vessel was statistically significant in such patients than those who remained in sinus rhythm with (*p* value = .046) as shown in (Table 2).

**Table 2:** Basal characteristics, echocardiographic data, angiographic results, and major adverse cardiac events in PIS subgroups

	Group IA (Patients with SR)	Group IB (Patients with NOAF)	*p* value
Age, years	61.91 ± 9.45	66.68 ± 7.75	.015*
Male gender, *n* (%)	135 (55.3%)	13 (52%)	.750
Smoking, *n* (%)	38(15.6%)	8 (32%)	.038*
Hypertension, *n* (%)	80 (32.8%)	14 (56%)	.020*
Diabetes mellitus, *n* (%)	99 (40.6%)	9 (36%)	.657
Dyslipidemia, *n* (%)	111 (45.5%)	8 (32%)	.196
Prior MI, *n* (%)	19 (7.8%)	4 (16%)	.162
LVEF, (%)	46.71 ± 3.77	42.48 ± 4.87	.001*
LAVI, ml/m^2^	33.60 ± 1.41	34.72 ± 1.77	.001*
E/e’ ratio	11.22 ± 0.98	13.01 ± 2.25	.001*
LM coronary artery, *n* (%)	3 (1.1%)	1 (4%)	.276
LAD coronary artery, *n* (%)	99 (40.6%)	7 (28%)	.220
CX coronary artery, *n* (%)	68 (27.9%)	6 (24%)	.680
Right coronary artery, *n* (%)	62 (25.4%)	11 (44%)	.046*
Mortality, *n* (%)	6 (2.5%)	1 (4%)	.645
Cardiogenic shock, *n* (%)	15 (6.1%)	3 (12%)	.265
Heart failure, *n* (%)	18 (7.4%)	4 (16%)	.134
Major bleeding, *n* (%)	6 (2.5%)	2 (8%)	.120
Reinfarction, *n* (%)	4 (1.6%)	4 (16%)	.405
Cerebral stroke, *n* (%)	2 (0.8%)	1 (4%)	.149

* indicates significant *p* value.

Abbreviations: CX, circumflex; LAD, left anterior descending; LAVI, left atrial volume index; LM, left main; LVEF, left ventricular ejection fraction; MI, myocardial infarction; NOAF, new‐onset atrial fibrillation; SR, sinus rhythm.

Subgroup analysis of group II (PPCI group), patients with NOAF (Subgroup IIB) were also older in age than patients who remained in sinus rhythm (Subgroup IIA). History of hypertension and prior myocardial infarction were predominant in such group. As regarding echocardiographic finding, patients in subgroup IIB had a lower LVEF, higher E/e’ ratio, and higher LAVI than patients who remained in sinus rhythm. RCA as a culprit vessel was more predominant in patients with NOAF in this group with (*p* value = .002). Moreover, cardiogenic shock and heart failure occurred more frequently in subgroup IIB than those who remained in sinus rhythm as shown in (Table 3). We included all risk factors that may affect the occurrence of NOAF in all patients of both groups in Table 4. The variables that were significantly affecting the outcome by bivariate analysis were included in multivariate analysis. The effect of treatment strategy (PIS vs. PPCI) on NOAF was tested by univariate analysis, and the *p* value was not significant (*p* value = .969). So, we did not include it in multivariate analysis.

**Table 3:** Basal characteristics, echocardiographic data, angiographic results, and major adverse cardiac events in PPCI subgroups

	Group IIA (Patients with SR)	Group IIB (Patients with NOAF)	*p* value
Age, years	61.73 ± 9.62	67.42 ± 9.82	.006*
Male gender, *n* (%)	133(56.1%)	17 (70.8%)	.165
Smoking, *n* (%)	58 (24.5%)	3 (12.5%)	.187
Hypertension, *n* (%)	57 (24.1%)	15 (62.5%)	.001*
Diabetes mellitus, *n* (%)	102 (43%)	10 (41.7%)	.845
Dyslipidemia, *n* (%)	89 (37.6%)	13 (54.2%)	.112
Prior MI, *n* (%)	26 (11%)	7 (29.2%)	.011*
LVEF, (%)	45.90 ± 5.99	43.13 ± 7.49	.037*
LAVI, ml/m^2^	33.37 ± 1.94	34.75 ± 2.34	.001*
E/e’ ratio	11.50 ± 1.40	12.33 ± 1.07	.006*
LM coronary artery, *n* (%)	4 (1.7%)	0 (0%)	.521
LAD coronary artery, *n* (%)	112 (47.3%)	7 (29.2%)	.090
CX coronary artery, *n* (%)	81 (34.2%)	5 (20.8%)	.185
Right coronary artery, *n* (%)	51 (21.5%)	12 (50%)	.002*
Mortality, *n* (%)	5 (2.1%)	0 (0%)	.472
Cardiogenic shock, *n* (%)	23 (9.7%)	6 (25%)	.023*
Heart failure, *n* (%)	14 (5.9%)	7 (29.2%)	.001*
Major bleeding, *n* (%)	3 (1.3%)	1 (4.2%)	.270
Reinfarction, *n* (%)	6 (2.5%)	2 (8.3%)	.116
Cerebral stroke, *n* (%)	3 (1.3%)	1 (4.2%)	.270

* indicates significant *p* value.

Abbreviations: CX, circumflex; LAD, left anterior descending; LAVI, left atrial volume index; LM, left main; LVEF, left ventricular ejection fraction; MI, myocardial infarction; NOAF, new‐onset atrial fibrillation; SR, sinus rhythm.

**Table 4:** Basal characteristics, echocardiographic data, angiographic results, and major adverse cardiac events in all patients of both groups with and without NOAF

	(Patients with SR) (*N* = 481, 90.8%)	(Patients with NOAF) (*N* = 49, 9.2%)	*p* value
Age, years	61.82 ± 9.52	67.04 ± 8.73	.001*
Male gender, *n* (%)	268(55.7%)	30 (61.2%)	.459
Smoking, *n* (%)	96 (20.0%)	11 (22.4%)	.679
Hypertension, *n* (%)	137 (28.5%)	29 (59.2%)	.001*
Diabetes mellitus, *n* (%)	201 (41.8%)	19 (38.8%)	.870
Dyslipidemia, *n* (%)	200 (41.6%)	21 (42.9%)	.863
Prior MI, *n* (%)	45 (9.4%)	11 (22.4%)	.005*
LVEF, (%)	46.31 ± 5.01	42.80 ± 6.23	.001*
LAVI, ml/m^2^	33.48 ± 1.69	34.73 ± 2.05	.001*
E/e’ ratio	11.36 ± 1.22	12.67 ± 1.78	.001*
LM coronary artery, *n* (%)	7 (1.5%)	1 (2.0%)	.749
LAD coronary artery, *n* (%)	211 (43.9%)	14 (28.6%)	.039*
CX coronary artery, *n* (%)	149 (31.0%)	11 (22.4%)	.215
Right coronary artery, *n* (%)	113 (23.5%)	23 (46.9%)	.001*
Mortality, *n* (%)	11 (2.3%)	1 (2.0%)	.912
Cardiogenic shock, *n* (%)	38 (7.9%)	9 (18.4%)	.014*
Heart failure, *n* (%)	32 (6.7%)	11 (22.4%)	.001*
Major bleeding, *n* (%)	9 (1.9%)	3 (6.1%)	.057
Reinfarction, *n* (%)	10 (2.1%)	3 (6.1%)	.081
Cerebral stroke, *n* (%)	5 (1.0%)	2 (4.1%)	.076

* indicates significant *p* value.

Abbreviations: CX, circumflex; LAD, left anterior descending; LAVI, left atrial volume index; LM, left main; LVEF, left ventricular ejection fraction; MI, myocardial infarction; NOAF, new‐onset atrial fibrillation; SR, sinus rhythm.

**Figure 1:**
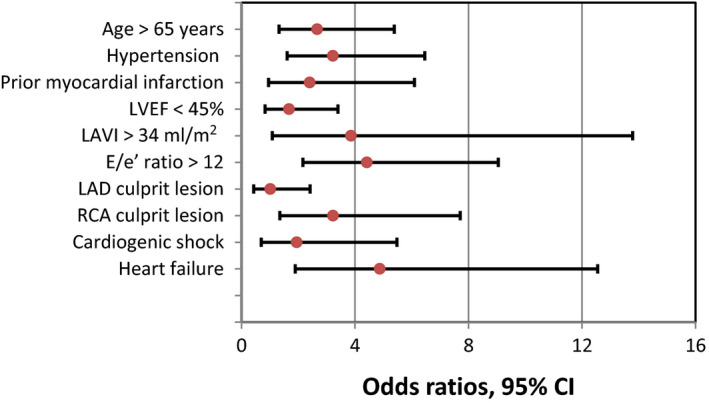
Forest plot of the multivariate regression analysis showing odds ratios, 95%CI of predictors of new‐onset atrial fibrillation

Multivariate regression analysis was performed to identify the independent predictors of NOAF as shown in Table 5and Figure 1; age ˃65 years [OR = 2.67, 95% CI = 1.32–5.38, *p* = .006], hypertension [OR = 3.22, 95% CI = 1.60–6.45, *p* = .001], LAVI ˃34 ml/m^2^ [OR = 3.86, 95% CI = 1.08–13.78, *p* = .037], E/e’ ratio ˃12 [OR = 4.42, 95% CI = 2.16–9.04, *p* = .001], RCA culprit vessel[OR = 3.22, 95% CI = 1.35–7.71, *p* = .008], and heart failure [OR = 4.87, 95% CI = 1.89–12.55, *p* = .001] were the independent predictors of NOAF.

**Table 5:** Multivariate regression analysis showing the independent predictors of NOAF

	Multivariate regression analysis OR (95% CI)	*p* value
Age ˃65 years	2.67 (1.32–5.38)	.006*
Hypertension	3.22 (1.60–6.45)	.001*
Prior myocardial infarction	2.40 (0.95–6.09)	.063
LVEF ˂45%	1.68 (0.83–3.40)	.150
LAVI ˃34 ml/m^2^	3.86 (1.08–13.78)	.037*
E/e’ ratio ˃12	4.42 (2.16–9.04)	.001*
LAD culprit lesion	1.01 (0.43–2.41)	.966
RCA culprit lesion	3.22 (1.35–7.71)	.008*
Cardiogenic shock	1.94 (0.69–5.47)	.206
Heart failure	4.87 (1.89–12.55)	.001*

* indicates significant *p* value.

Abbreviations: CI, confidence interval; LAD, left anterior descending; LAVI, left atrial volume index; LVEF, left ventricular ejection fraction; OR, odds ratio; RCA, right coronary artery.

## 4 DISCUSSION

The occurrence of NOAF in the setting of AMI has its detrimental effects on the outcome of such patients. The current study was conducted to investigate the incidence, independent predictors of NOAF and to assess the outcome of NOAF. Over a period of nearly two years, we studied a total of 530 patients with STEMI who presented to our department. The incidence of NOAF was nearly similar in both groups (9.3% in group I vs. 9.2% in group II, *p* value = .969). Another important finding was that, the right coronary artery was the commonly affected vessel in patients who developed NOAF. This can be explained by the development of atrial and sinus node ischemia due to impairment of blood supply in the sinus node artery or atrioventricular node artery causing ischemia of atrial wall and sinus node. Atrial ischemia and right atrial volume overload due to right ventricular myocardial infarction were postulated to be pathophysiologic mechanisms for NOAF in occlusion of right coronary artery (Goldstein, 2002). Moreover, increased left atrial pressure due to left ventricular dysfunction and increased vagal stimulation has been also postulated to be another possible mechanisms of infarct associated NOAF.

Our study investigated the independent predictors of NOAF, multivariate regression analysis identified the predictors of NOAF that were (advanced age ˃65 years, history of hypertension, left atrial volume index (LAVI) ˃34 ml/m^2^, E/e’ ratio ˃12 and presence of heart failure. In agreement to our results Vukmirović et al. (2017) studied the predictors and outcomes of NOAF in patients with AMI and found that, the predictors for occurrence of NOAF were old age and obesity. They found such patients had a higher mortality than patients who remained in sinus rhythm. Death, recurrent infarction, revascularization, and stroke occurred more frequent in patients who developed NOAF than patients who remained in sinus rhythm. The incidence of NOAF was found to be 8%, they included both STEMI and non‐STEMI patients. However, in our study mortality, recurrent infarction and cerebral stroke showed no statistically significant difference between patients with NOAF and patients who remained in sinus rhythm.

GUSTO I trial (Crenshaw et al., 1997) showed patients who developed NOAF in subjects who presented with STEMI and received fibrinolytic therapy were likely to be older, female, and higher Killip class. They also were more likely to have diabetes mellitus, hypertension, and lower LVEF than patients who remained in sinus rhythm. The study found mortality to be higher in patients who developed NOAF. Their results came in agreement with the results of the present study in advanced age of the patients, presence of hypertension as a risk factor and lower LVEF in patients with NOAF. Rhyou et al. (2018) studied the clinical factors associated with the development of NOAF in the year following STEMI treated by PPCI. The incidence of NOAF was found to be 15.4% which was higher than the incidence of NOAF in this study. They found patients who developed NOAF were older, female, with congestive heart failure. They experienced cardiogenic shock, had lower LVEF, higher E velocity, E/e’ ratio, and LAVI than patients who remained in sinus rhythm. The results were similar to the results of the present study. Mrdovic et al. (2012) studied the incidence, predictors, and 30‐day outcomes of NOAF after primary PCI. They found 6.2% of patients developed NOAF. They also found older age of the patient, Killip class heart failure more than 1, preprocedural infarct‐related artery occlusion and postprocedural TIMI flow <3 were independent risk factors for the occurrence of NOAF.

## 5 CONCLUSION

The current study investigated patients with STEMI subjected to reperfusion either by PIS or PPCI and found that, the incidence of NOAF was nearly similar in the two strategies of treatment. The independent predictors of NOAF identified by the multivariate regression analysis were (advanced age ˃65 years, history of hypertension, left atrial volume index (LAVI) ˃34 ml/m^2^, E/e’ ratio ˃12, RCA culprit vessel, and presence of heart failure). Moreover, the short‐term outcomes including mortality, reinfarction, and cerebral stroke were similar in both groups.

## CONFLICT OF INTEREST

None declared.

## 6 AUTHOR CONTRIBUTIONS

Conceived and designed the analysis: MK, AE. Collected the data: MK, AE. Contributed data or analysis tools: MK, AE. Performed the analysis: MK. Wrote this paper: MK.

## ETHICS

All procedures performed in this study were in accordance with the ethical standards of the institutional and national research committee and with the 1964 Helsinki declaration and its later amendments.
